# IgM, IgG, and IgG Subclass Antibody Responses to *Plasmodium falciparum* Proteins in Naïve, Malaria-Vaccinated and Semi-Immune Volunteers after Controlled Human Malaria Infection

**DOI:** 10.4269/ajtmh.25-0384

**Published:** 2025-09-30

**Authors:** Gloria P. Gómez-Pérez, Marta Vidal, Joseph J. Campo, Gemma Moncunill, Alfons Jimenez, Miquel Vázquez-Santiago, Gemma Ruiz-Olalla, Héctor Sanz, Aintzane Ayestaran, Evelina Angov, Sheetij Dutta, Chetan Chitnis, Virander Chauhan, Eric R. James, Peter F. Billingsley, B. Kim Lee Sim, Peter G. Kremsner, Stephen L. Hoffman, Bertrand Lell, Benjamin Mordmüller, Carlota Dobaño

**Affiliations:** ^1^ ISGlobal, Barcelona, Spain;; ^2^ Facultat de Medicina i Ciències de la Salut, Universitat de Barcelona (UB), Barcelona, Spain;; ^3^ CIBER de Enfermedades Infecciosas (CIBERINFEC), Barcelona, Spain;; ^4^ CIBER de Epidemiología y Salud Pública (CIBERESP), Barcelona, Spain;; ^5^ Walter Reed Army Institute of Research (WRAIR), Biologics Research & Development Branch, Silver Spring, Maryland;; ^6^ International Centre for Genetic Engineering and Biotechnology (ICGEB), New Delhi, India;; ^7^ Sanaria Inc, Rockville, Maryland;; ^8^ Centre de Recherches Médicales de Lambaréné (CERMEL), Albert Schweitzer Hospital, Lambarene, Gabon;; ^9^ Institute of Tropical Medicine and German Center for Infection Research, University of Tübingen, Tübingen, Germany;; ^10^ Division of Infectious Diseases and Tropical Medicine, Department of Medicine I, Medical University of Vienna, Vienna, Austria;; ^11^ Department of Medical Microbiology, Radboud University Medical Center, Nijmegen, The Netherlands

## Abstract

The immune response to malaria vaccines is generally stronger in malaria-naive individuals than in those with lifelong exposure. The immunological basis for this is unclear. IgM, total IgG, and IgG subclass (IgG1, IgG2, IgG3, and IgG4) antibody responses against 21 pre-erythrocytic and erythrocytic *Plasmodium falciparum* proteins before and after controlled human malaria infection (CHMI) via the direct venous inoculation of 3,200 *P. falciparum* sporozoites (PfSPZ) were compared in three groups of volunteers: 1) malaria-naïve (*n* = 22); 2) malaria-naïve immunized with a PfSPZ chemoattenuated vaccine (PfSPZ-CVac) (*n* = 27); and 3) lifelong malaria-exposed individuals from Africa (*n* = 20), including those with normal hemoglobin (*n* = 11) or sickle cell trait (*n* = 9). Before and after CHMI, PfSPZ-CVac-immunized individuals exhibited higher levels of IgM and IgG to CSP and SSP-2/TRAP than the other two groups. Malaria-experienced Africans exhibited more intense and broader antibody responses to blood-stage (BS) antigens than naïve and vaccinated individuals, longer pre-patent periods (PPPs), and fewer symptoms. Among confirmed malaria cases, cytophilic IgG1 and IgG3 antibodies to BS antigens were positively associated with longer PPPs, whereas IgG2, IgG4, and IgM were not. IgG2 and IgG4 (noncytophilic) *P. falciparum*-specific antibodies were higher in the semi-immune group, including elevated anti-CSP IgG4 (regulatory) levels. The IgM response in African volunteers post-CHMI was stronger than that in malaria-naïve and vaccinated individuals and had the hallmark of a secondary memory response. Cytophilic immunoglobulins controlled parasitaemia better than noncytophilic immunoglobulins. However, elevation of the latter in lifelong malaria-exposed individuals could be associated with regulatory responses and hamper vaccine efficacy.

## INTRODUCTION

According to the latest World Malaria Report, in 2023, there were 263 million cases of malaria globally, representing an increase of 11 million compared with 2022.^1^ Furthermore, political conflict and humanitarian crises, drug and insecticide resistance, and climate change, pose challenges to controlling malaria in the coming years. The world’s first approved malaria vaccine, RTS,S/AS01_E_,^2^ administered to young children in Ghana, Kenya, and Malawi, led to a 32% reduction in hospital admission and a 9% reduction in all-cause mortality.^3^ However, sustained immune response may require a booster dose more frequently than annually. The second approved malaria vaccine, R21/Matrix-M, has exhibited promising efficacy^4^ and is also being administered in Africa. However, these subunit vaccines have efficacies well below the 75% strategic goal of the WHO and need to demonstrate protection over ≥2 years.^5^ In contrast, a high level of protection (>80%) against infection with *Plasmodium falciparum* has been achieved in malaria-naïve volunteers via immunization with radiation-attenuated sporozoites,^6^^,^^7^ genetically attenuated sporozoites,^8^^–^^10^ or inoculation of infectious *P. falciparum* sporozoites (PfSPZ) in volunteers taking antimalarial chemoprophylaxis.^11^^–^^13^ Nevertheless, the efficacy of the irradiated PfSPZ vaccine in healthy adults with lifelong exposure to malaria in Africa (semi-immune) is generally lower than in malaria-naïve individuals undergoing similar vaccine regimens,^14^^–^^18^ and this observation also applies to other malaria vaccines. The immune mechanisms causing these significant differences in vaccine efficacy between semi-immune versus malaria-naïve populations remain elusive.

In contrast to the sterile immunity induced by PfSPZ vaccines,^7^^,^^13^ naturally acquired immunity (NAI) rarely provides infection-blocking protection to falciparum malaria and is mainly mediated by immunity to erythrocytic stage parasites.^19^ *Plasmodium falciparum*-specific antibodies and CD4^+^ T cells have been shown to play a critical role in NAI,^20^ although they are acquired slowly under natural conditions that are hypothetically hampered by malaria-induced immunosuppressive mechanisms.^21^^–^^23^ The type of antibody response triggered by pathogens depends on several factors, including the biochemical characteristics of the antigens (polysaccharides, lipids, and proteins), as well as the secondary signals they trigger in the immune system (effector or regulatory). Prototypically, protein antigens mainly engage B cells that receive T cell help (T cell-dependent [TD] antibody response), performing a class switch to immunoglobulin (Ig) G3, IgG1, IgG2, and IgG4 (in this order),^24^ and also to IgA1 and IgE,^25^ generating memory B cells (MBCs) and, in some cases, long-lived plasma cells. However, the human immune system can also respond without T cell help (T cell-independent [TI] antibody response) to protein and TD antigens,^26^ in a similar fashion as to polysaccharides, inducing the rapid production of specific IgM antibodies, class switch to IgG2, IgA2, and even IgG1,^25^^,^^27^ and generating short-lived plasma cells and IgM^+^ MBCs at first encounter (primary response). Moreover, in the case of the humoral response to falciparum malaria, the TI and TD origin of *P. falciparum*-specific IgG subclasses remains to be characterized in more detail. Currently, there is increasing evidence that lifelong exposure to malaria parasites triggers a long-lasting IgM-mediated immunity,^28^^–^^34^ although the mechanisms and underlying evolutionary processes of such a response are poorly understood.

T cell-independent IgM can be readily produced within 48 h after encountering an antigen^26^ and activates the classical complement cascade with a 1,000-fold higher avidity than IgG.^29^ Additionally, IgM binds with high affinity to the human IgM-Fc receptors FcμR and Fcα/μR on B cells, T cells, NK cells, follicular dendritic cells, and macrophages.^35^^,^^36^ Through these receptors, IgM regulates B cell activation and B cell-mediated T cell immunity and triggers antibody-dependent phagocytosis of pathogens.^36^ Furthermore, although IgG subclasses are >90% identical at the molecular level, each is present at a different concentration in peripheral blood (in order of decreasing abundance: IgG1, IgG2, IgG3, and IgG4) and has a unique structure and function.^25^ For instance, IgG2 and IgG4 are considered regulatory, noncytophilic antibodies because they bind fewer members of the Fc receptor family in immune cells and with lower avidity than IgG1 and IgG3 (cytophilic antibodies).^25^ IgG2 and IgG4 are not potent complement activators, whereas IgG1 and IgG3 trigger antibody-dependent cellular phagocytosis and have a more efficient complement-dependent cytotoxicity.^24^^,^^25^^,^^37^ In addition, switching to IgG4 may be modulated by interleukin (IL)-10 (a regulatory cytokine), linking this subclass with the induction of tolerance.^25^ Studies have revealed that the correlation between levels of noncytophilic antibodies to *P. falciparum* merozoites and Fc-mediated functional assays is less compared with cytophilic antibodies, indicating that the Fc-mediated effector functions triggered differ between IgG subclasses, with possible implications on parasitological and clinical outcomes.^38^

Most studies of anti-falciparum malaria antibodies focus on TD responses (i.e., assessing only total IgG, infrequently IgM, and rarely IgG subclasses found at lower plasma concentrations). Consequently, limited and contradictory data are available on the role of IgG2 and IgG4 in protection against *P. falciparum* infection and symptomatic malaria.^39^^–^^41^ Remarkably, IgM antibodies and IgM^+^ MBCs play an essential role in clinical malaria protection in chronically exposed individuals.^28^^,^^30^^,^^31^ Additionally, PfSPZ vaccine-induced anti-CSP IgM antibodies from semi-immune individuals have been shown to block PfSPZ invasion of hepatocytes in vitro,^34^ as well as merozoite invasion of erythrocytes in a complement-dependent manner.^28^

Falciparum malaria parasites express hundreds of different proteins and antigens from their complex array of about 5,000 genes^5^ during their pre-erythrocytic and asexual blood stages (BSs). Therefore, it is important to study the humoral response to both falciparum malaria stages in the human host to understand which Ig bound to which antigens can confer protection against infection and disease, or instead trigger a regulatory response that may hamper the control of infection and vaccine immunogenicity. This knowledge can aid in identifying the right targets for malaria vaccines linked to strong antibody effector functions, thereby improving vaccine efficacy and counteracting immune evasion mechanisms.

**Figure 1.**
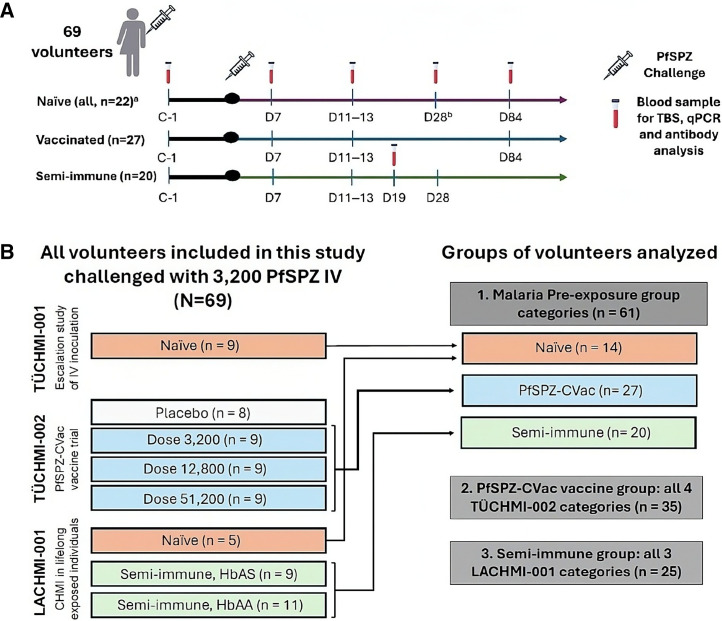
(**A**) Experimental design. ^a^This sample size represents all naïve volunteers, including the placebo group from the PfSPZ-CVac study. ^b^Blood samples for antibody analysis were not taken at this time point from the control volunteers from the PfSPZ-CVac study. (**B**) Distribution of volunteers from the three different CHMI studies into the comparison groups analyzed. C-1 = 1–2 days before CHMI; CHMI = controlled human malaria infection; D7, D11–13, D28, and D84 = 7, 11–13, 28, and 84 days after CHMI, respectively; HbAA = normal hemoglobin; HbAS =sickle cell-trait hemoglobin; PfSPZ = *Plasmodium falciparum* sporozites; PfSPZ-CVac = PfSPZ chemoattenuated vaccine; PfSPZ challenge = intravenous inoculation of 3,200 PfSPZ, NF54 strain; qPCR = quantitative polymerase chain reaction; TBS = thick blood smear.

Taking advantage of the highly standardized conditions of controlled human malaria infection (CHMI) studies, both the TD and TI antibody responses to *P. falciparum* challenge were investigated by measuring the levels of IgM, IgG, and IgG1−IgG4 against multiple pre-erythrocytic and erythrocytic proteins before and after the direct venous inoculation (DVI) of PfSPZ. Their association with sterile protection, pre-patent period, and clinical malaria was evaluated in 69 volunteers from three different CHMI studies analyzed in three comparison groups: 1) a malaria pre-exposure group (*n* = 61); 2) a PfSPZ chemoattenuated vaccine (PfSPZ-CVac) group (*n* = 35); and 3) a semi-immune group (*n* = 25; Figure 1).

## MATERIALS AND METHODS

### Study population.

Samples were obtained from 69 healthy adult volunteers participating in two CHMI trials conducted in Tübingen, Germany (TÜCHMI-001, *n* = 9 and TÜCHMI-002, *n* = 35) and one CHMI trial conducted in Lambaréné, Gabon (LACHMI-001, *n* = 25; Table 1). Detailed information on the participants and the ethical approvals for the clinical and immunology studies has been published elsewhere.^13^^,^^42^^,^^43^ Briefly, all volunteers underwent CHMI via DVI of the same dose of 3,200 aseptic, purified, cryopreserved, infectious PfSPZ strain NF54, hereafter referred to as PfSPZ Challenge.^44^ The level of previous malaria exposure varied among participants in the three trials: TÜCHMI-001 was a PfSPZ dose-escalation CHMI study involving malaria-naïve volunteers^42^; TÜCHMI-002 was a PfSPZ-CVac vaccine trial in which malaria-naïve volunteers were inoculated intravenously (IV) with three different doses of PfSPZ (3,200; 12,800; 51,200; *n* = 9 each) or placebo (*n* = 13; only 8/13 included in this analysis) under chloroquine (CQ) prophylaxis followed by PfSPZ Challenge 8–10 weeks after the last dose of the vaccine (Table 1)^13^; and LACHMI-001 was a CHMI trial involving semi-immune African volunteers with normal (normal hemoglobin [HbAA], *n* = 11) or SCT (sickle cell-trait hemoglobin [HbAS], *n* = 9), as well as a control group of local, nonimmune Europeans without a history of malaria and no long-term residence in Gabon (<11 months, *n* = 5; Table 1).^43^ These trials are registered at ClinicalTrials.gov under numbers NCT01624961 (TÜCHMI-001), NCT02115516 (TÜCHMI-002), and NCT02237586 (LACHMI-001).

**Table 1:** Baseline demographic characteristics and controlled human malaria infection studies

Parameters and Groups of Volunteers	TÜCHMI-001* (*n =* 9)	TÜCHMI-002^†^ (*n =* 35)	LACHMI-001 (*n =* 25)	Total (*N* = 69)	Malaria, *n* (%)
Age median in years (range)	27 (24‒30)	26 (20–39)	23 (19‒29)	–	–
Sex, male (%)	7 (78)	18 (51)	13 (52)	38 (55)	–
Malaria Pre-exposure group	–	–	–	61	–
Naïve	9	–	5	14	14 (100)
PfSPZ-CVac	–	27	–	27	9 (33)
Semi-immune	–	–	20	20	16 (80)
Semi-immune group	–	–	–	25	–
Naïve	–	–	5	5	5 (100)
Semi-immune HbAA	–	–	11	11	9 (82)
Semi-immune HbAS	–	–	9	9	7 (78)
PfSPZ-CVac vaccine group	–	–	–	35	–
Placebo^‡^ + CQ	–	8	–	8	8 (100)
3,200 PfSPZ + CQ	–	9	–	9	6 (67)
12,800 PfSPZ + CQ	–	9	–	9	3 (33)
51,200 PfSPZ + CQ	–	9	–	9	0 (0)
Chemoprophylaxis	None	Chloroquine^§^	Clindamycin^‖^	–	–

CQ = chloroquine; HbAA = normal hemoglobin genotype; HbAS = sickle cell trait hemoglobin genotype; PfSPZ = *Plasmodium falciparum* sporozoites; PfSPZ-CVac = immunization via direct venous inoculation of aseptic, purified, cryopreserved, non-irradiated PfSPZ to malaria-naïve, healthy adult volunteers taking chloroquine for antimalarial chemoprophylaxis.

* TÜCHMI-001 was a dose-escalation study in which volunteers received intravenous injections of 50-, 200-, 800-, or 3,200-dose PfSPZ.^42^ In the present analysis, only the volunteers injected with the 3,200 PfSPZ dose were included.

^†^ TÜCHMI-002 samples for the present analysis were collected during the controlled human malaria infection study performed 8–10 weeks after the participants received the last PfSPZ-CVac immunization.

^‡^ The TÜCHMI-002 placebo group included 13 volunteers, of whom eight were randomly selected for the present analysis.^13^

^§^ Chloroquine was administered 2 days before the first dose of the vaccine, and weekly doses were administered until 5 days after the last dose of the vaccine. Malaria challenge was performed 7–9 weeks after the last dose of chloroquine.

^‖^ Clindamycin was administered for 5 days to all volunteers. Malaria challenge was performed 2 days after the last dose. Clindamycin targets asexual liver and blood-stage parasites and has a half-life of 2–4 hours; therefore, it does not interfere with subsequent malaria challenge.^43^

### Sample collection and diagnosis.

Blood samples were collected before and at several timepoints after CHMI for thick blood smear (TBS), quantitative polymerase chain reaction (qPCR), and immunology studies (Figure 1A). From day (D) 5 until antimalarial treatment, blood samples were withdrawn daily for TBS and qPCR analysis. Quantitative TBS (Lambaréné method) and qPCR were performed using published procedures for microscopic malaria diagnosis^45^ and the quantification of parasites, respectively.^13^^,^^42^^,^^43^ Thick blood smears were analyzed the same day as well as shortly after blood samples were taken, whereas qPCR analyses were performed after all follow-up visits were completed. Plasma samples were collected 1–2 days before CHMI (C-1), as well as on the following days after CHMI: 7 (D7), between 11 and 13 (D11–13), 19 (D19) only from the semi-immune individuals in LACHMI-001, 28 (D28) only in TÜCHMI-001 (naïve) and LACHMI-001 (semi-immune/controls), and 84 (D84) only in TÜCHMI-001 (naïve) and TÜCHMI-002 (vaccinated).

### Antibody measurement.

Antibodies against five pre-erythrocytic and 16 erythrocytic *P. falciparum* antigens, including 3D7 and FVO strains for two of them (Supplemental Table 1), were measured using quantitative suspension array technology with xMAP^™^ technology (Luminex Corp., Austin, TX), as previously described.^46^^–^^48^ Study samples were tested in duplicate at 1/500 and 1/20,000 for IgG, 1/400 and 1/12,000 for IgG1, 1/50 for IgG2 and IgG4, 1/200 and 1/1,000 for IgG3, and 1/200 and 1/20,000 for IgM. The distribution of test samples and positive and negative controls was balanced across the plates. Briefly, *P. falciparum* protein antigens were covalently coupled directly to the beads and blocked with BSA; 1,000 microspheres were used per analyte and sample. A plasma pool from falciparum malaria hyperimmune Mozambican adult volunteers was used as a positive control in each plate. Samples from malaria-naïve European adults were used as negative controls. Two wells per plate were assayed without plasma added as blank controls. Samples or blanks were first incubated with the antigen-coupled beads, next with biotinylated anti-human secondary antibodies, and finally with streptavidin-conjugated R-phycoerythrin. Antibody levels were quantified as median fluorescence intensity (MFI) using a Luminex 200^™^ (Luminex Corp.) analyzer and Xponent software version 3.1 (Luminex Corp.), with at least 50 microspheres per analyte acquired per sample. Additional information about the antibody assays can be found in the Supplemental Information.

### Statistical methods and study design.

Data preprocessing of MFI values is described in the Supplemental Information. Log_10_-transformed MFI values were analyzed in 69 volunteers, both before and after CHMI, within the three comparison groups listed below (Figure 1B).

#### Group 1: Malaria pre-exposure group.

Sixty-one volunteers were classified into three categories: naïve individuals experiencing their first infection (*n* = 14, from TÜCHMI-001 [*n* = 9] and LACHMI-001 controls [*n* = 5]); PfSPZ-CVac-vaccinated individuals with three different doses of chemoattenuated parasites who experienced very low parasitemia two or three times (*n* = 27, from TÜCHMI-002); and semi-immune individuals (*n* = 20, lifelong malaria-exposed volunteers from LACHMI-001). The placebo group from TÜCHMI-002 was not included in the malaria-naïve category for this analysis because they received CQ prophylaxis (an antimalarial with recognized immunoregulatory properties),^49^ although at the time of CHMI, CQ concentrations were expected to be below therapeutic ranges.

#### Group 2: PfSPZ-CVac vaccine group.

Thirty-five volunteers were classified into four categories: three groups that received varying PfSPZ-CVac vaccine doses (*n* = 9 each) and the placebo group (*n* = 8).

#### Group 3: Semi-immune group.

Twenty-five volunteers were classified into three categories: semi-immune with HbAS (*n* = 9), semi-immune with HbAA (*n* = 11), and naïve controls (all HbAA, *n* = 5). Normality was evaluated using the Shapiro–Wilk test, followed by two types of analyses.

#### Comparison of antibody levels among categories within each comparison group (malaria pre-exposure, PfSPZ-CVac vaccine, and semi-immune groups).

Differences in antibody log_10_ MFI values and fold-changes from C-1 to D7, D11–13, and D28 (timepoint after CHMI/C-1) were analyzed. Fold-changes were calculated as the log_10_ MFI antibody levels at the timepoint after the CHMI/log_10_ MFI antibody levels at C-1. Differences were analyzed using Kruskal–Wallis rank sum and Dunn’s tests (nonparametric pairwise multiple comparison test).

#### Association of antibody levels with protection against malaria.

This analysis was performed only in the following groups and volunteers:

##### Malaria pre-exposure group.

Univariable and multivariable logistic and linear regression models to analyze the associations between antibody responses (log_10_MFI levels or fold-changes as independent continuous variables) and the following outcomes: malaria infection (by TBS or qPCR or binary dependent variables) and days between CHMI and the first positive malaria test result (pre-patent period by TBS and time to first positive qPCR, dependent continuous variables). Covariates were sex and malaria pre-exposure status (category), with naïve individuals as controls.

##### Lifelong malaria-exposed individuals (semi-immune with HbAA and semi-immune with HbAS).

Univariate and multivariate logistic and linear regression models to analyze the associations between antibody responses (log_10_ MFI levels or fold changes as independent continuous variables) and symptomatic malaria (a binary dependent variable), with sex as a covariate.

*P*-values were considered statistically significant when they were <0.05 after adjustment for testing of multiple antigens using the Benjamini–Hochberg method, and they were considered trends when they were ≥0.05 and <0.1. All data collected were preprocessed, managed, and analyzed using R software version 4.0.3 (R Foundation, Vienna, Austria).

## RESULTS

### Comparison of antibody levels between categories within each comparison group.

#### Antibody response in the malaria pre-exposure group.

##### Before CHMI (timepoint C-1).

**Figure 2.**
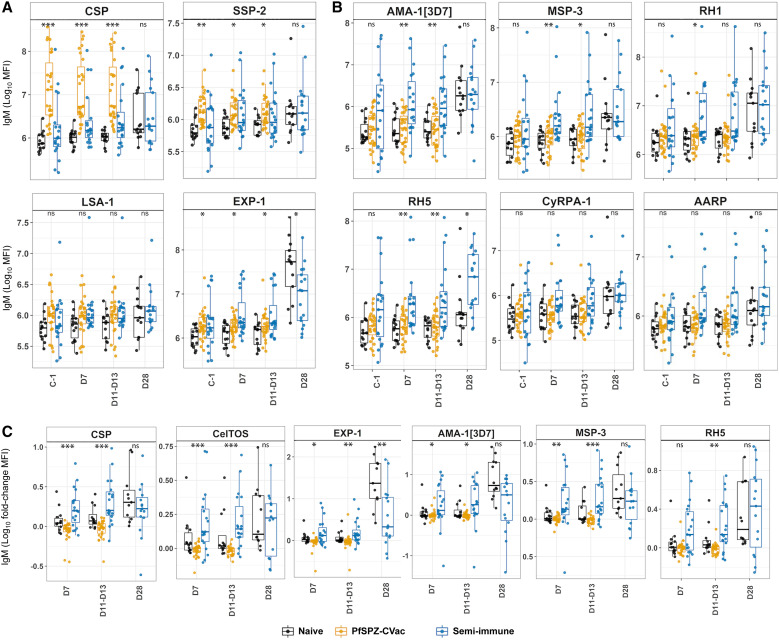
IgM response in naïve, PfSPZ-CVac, and semi-immune volunteers before and after PfSPZ challenge. (**A**) Magnitude of IgM response to sporozoite pre-erythrocytic antigens measured in log_10_ MFI. (**B**) Magnitude of IgM response to erythrocytic antigens. (**C**) Fold-change in IgM levels to selected pre-erythrocytic and erythrocytic antigens. *y*-axis, log_10_ MFI (**A** and **B**) and fold-change of MFI (**C**). *x*-axis, timepoints studied: C-1; D7; D11–D13; D28. Boxplots: lower and upper lines represent Q1 and Q3 and show the IQRs (IQR = Q3–Q1); horizontal lines within boxes indicate medians (Q2); whisker lines correspond to the highest and lowest values no greater than 1.5 times the IQR; and dots represent individual data points. Data points beyond the whisker lines are outliers. *P*-values of the Kruskal–Wallis test used to compare the naïve, PfSPZ-CVac and semi-immune volunteers are shown as follows: *P-value <0.05; **P-value <0.01; ***P-value <0.001. Results of post hoc Dunn’s pairwise multiple comparison tests are presented in Supplemental Table 2. C-1 = 1–2 days before PfSPZ challenge; D7, D11–13, and D28 = 7, 11–13, and 28 days after PfSPZ challenge, respectively; IQR = interquartile range; log_10_ = logarithmic 10 scale; MFI = median fluorescence intensity; ns = no statistically significant difference; PfSPZ = *Plasmodium falciparum* sporozites; PfSPZ-CVac = PfSPZ chemoattenuated vaccine; Q = quartile.

**Figure 3.**
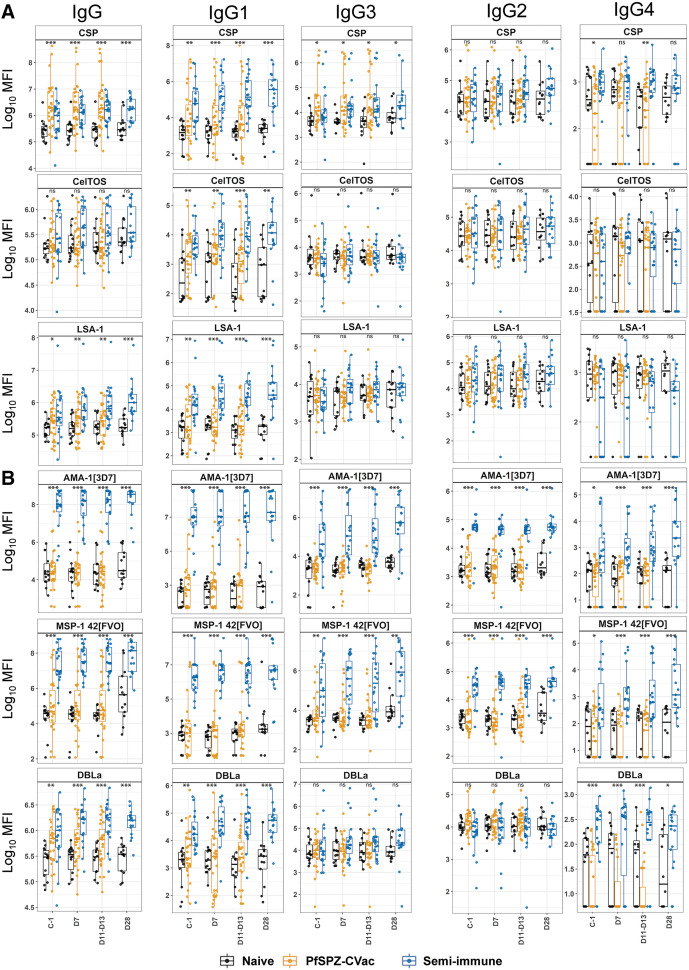
IgG and IgG isotype response in naïve, PfSPZ-CVac, and semi-immune volunteers (malaria pre-exposure group) before and after PfSPZ challenge. (**A**) Levels (log_10_ MFI) of IgG and IgG1–4 to selected pre-erythrocytic antigens. (**B**) Levels of IgG and IgG1–4 to selected pre-erythrocytic and erythrocytic antigens. *y*-axis, log_10_ MFI. *x*-axis, time points studied: C-1; D7; D11–D13; D28. Boxplots: lower and upper lines represent Q1 and Q3 and show the IQRs (IQR = Q3–Q1); horizontal lines within boxes indicate medians (Q2); whisker lines correspond to the highest and lowest values no greater than 1.5 times the IQR; and dots represent individual data points. Data points beyond the whisker lines are outliers. *P*-values of the Kruskal–Wallis test used to compare the naïve, PfSPZ-CVac, and semi-immune volunteers are shown as follows: *P-value <0.05; **P-value <0.01; ***P-value <0.001. Results of post hoc Dunn’s multiple comparison tests are presented in Supplemental Table 2. C-1 = 1–2 days before PfSPZ challenge; D7, D11–13, and D28 = 7, 11–13, and 28 days after PfSPZ challenge, respectively; IQR = interquartile range; log_10_ = logarithmic 10 scale; MFI = median fluorescence intensity; ns = no statistically significant difference; PfSPZ = *Plasmodium falciparum* sporozites; PfSPZ-CVac = PfSPZ chemoattenuated vaccine; Q = quartile.

Participants who had been vaccinated with PfSPZ-CVac (CQ) 10 weeks earlier exhibited significantly higher IgM levels to pre-erythrocytic antigens CSP and SSP-2/TRAP than semi-immune and naïve individuals, consistent with recent exposure to PfSPZ in the vaccinated group. They also exhibited higher IgM levels to EXP-1 (expressed by liver and asexual BS)^50^^,^^51^ than the malaria-naïve participants (Figure 2A, Table S2). At C-1, the semi-immune had higher IgM levels only to RH5 compared to the naïve, even though 4 of 20 semi-immune volunteers had asymptomatic parasitemia shortly before CHMI (i.e., they were naturally infected recently; Figure 2B; Supplemental Table 2). Levels of total IgG to 6/21 proteins (CSP, RH1, RH5, MSP-3, EBA-175, and DBLα) and IgG3 to CSP were also significantly higher in the PfSPZ-CVac volunteers than in the naïve volunteers. Compared with semi-immune individuals, the PfSPZ-CVac participants exhibited higher IgG levels to RH1, a trend of higher IgG levels to CSP, and higher IgG4 levels to EBA-175 and CyRPA-2 before CHMI (Supplemental Table 2). There were no significant differences in IgG1, IgG2, and IgG4 levels between the PfSPZ-CVac and naïve volunteers at baseline. Compared with PfSPZ-CVac and naïve individuals, semi-immune individuals at C-1 had significantly higher levels of total IgG (13/21 antigens), IgG1 to all studied antigens (21/21), IgG3 (9/20), and IgG4 (6/18), predominantly to erythrocytic antigens but also to pre-erythrocytic antigens, including higher IgG4 levels to CSP compared with the PfSPZ-CVac volunteers (Figure 3; Supplemental Table 2).

##### After CHMI (D7, D11–D13, and D28).

Individuals who received the PfSPZ-CVac maintained significantly higher IgM levels to CSP and SSP-2/TRAP compared with the naïve group (Figure 2A), but naïve individuals exhibited significantly higher IgM fold-changes to CSP, SSP-2/TRAP, and CelTOS compared with PfSPZ-CVac volunteers (Supplemental Table 3). The latter also exhibited significantly higher IgG levels to 5/21 antigens (CSP, RH1, RH5, EBA-175, and DBLα) compared with naïve individuals, although IgG fold-changes for some antigens were higher in the naïve group (Supplemental Table 3). No significant differences were observed between the PfSPZ-CVac and naïve groups in terms of IgG1, IgG2, IgG3, and IgG4 levels (Figure 3; Supplemental Figure 1; Supplemental Table 2). However, there was a trend toward higher IgG1 levels to CSP and RH5, as well as higher IgG3 levels to CSP, in the PfSPZ-CVac volunteers compared with the naïve volunteers at these time points.

**Figure 4.**
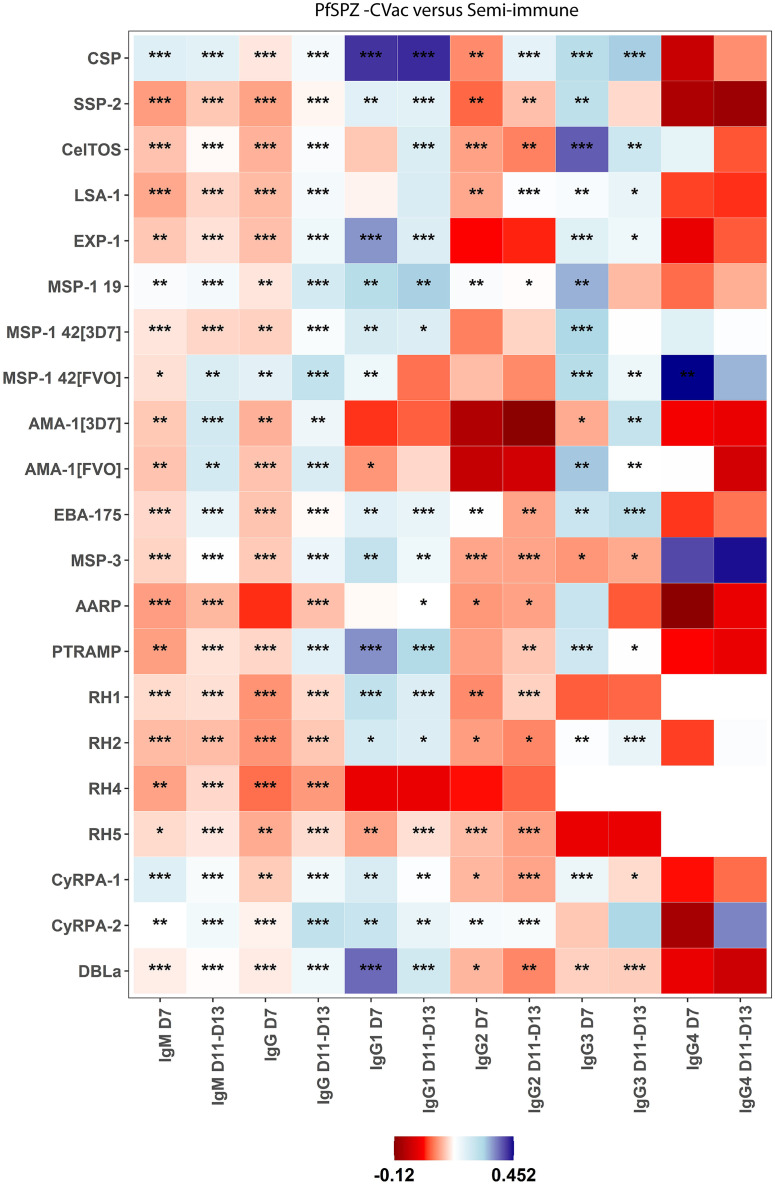
Heatmap analysis of the fold-change (FC) difference in antibody levels from baseline (1 to 2 days before *Plasmodium falciparum* sporozite [PfSPZ] challenge) to 7 days (D7) and 11–13 days (D11–13) after PfSPZ challenge between semi-immune, and PfSPZ chemoattenuated vaccine (PfSPZ-CVac) volunteers. Columns represent the FC difference in logarithmic 10 scale median fluorescence intensity between the two groups (FC semi-immune – FC PfSPZ-CVac) per isotype and timepoint (D7 or D11–13), and the rows represent the *P. falciparum* antigens studied. The FC differences are color coded from red (toward negative difference: FC semi-immune < FC PfSPZ-CVac) to blue (toward positive difference: FC semi-immune > FC PfSPZ-CVac); the white color (cut off for color coding) represents an FC difference = 0.166. The *P*-values of the Dunn test paired comparisons are shown as follows: *P-value ≥0.05 and <0.10; **P-value <0.05; ***P-value <0.01. Data are presented in Supplemental Table 3.

Semi-immune individuals exhibited higher IgM levels at D7 and D11–D13 than both the naïve and PfSPZ-CVac participants against almost all antigens, except for anti-CSP IgM levels that were higher in the PfSPZ-CVac individuals than in the semi-immune volunteers. These differences were statistically significant for 4/21 antigens (AMA-1_[3d7]_, EXP-1, RH5, and MSP-3), and a trend of higher IgM levels was found for 5/21 antigens (CSP, RH1, RH2, MSP-1_42[FVO]_, AARP; Figure 2B; Supplemental Table 2). The semi-immune group also exhibited significantly higher IgM fold-changes (D11–D13/C-1) to all antigens (21/21) compared with the PfSPZ-CVac participants (Figures 2C and 4; Supplemental Table 3), and a trend for CyRPA-1, MSP-3, RH1, and AARP, compared with the naïve group (Figure 2C; Supplemental Table 3). At D28, naïve individuals exhibited statistically significantly higher IgM levels or fold-changes (D28/C-1) to EXP-1 and MSP-1_42[3d7/FVO]_ compared with the semi-immune individuals (Supplemental Tables 2 and 3), who exhibited significantly higher IgM levels only to RH5 compared with naïve individuals.

Regarding IgG, semi-immune individuals maintained higher antibody levels for a majority of antigens throughout the post-challenge period compared with both PfSPZ-CVac and naïve individuals (Supplemental Figures 2 and 3). Anti-CSP IgG levels were significantly higher in semi-immune individuals compared with naïve individuals after CHMI, and the largest median differences in IgG levels (>3 log_10_ MFI) were found for highly immunogenic antigens AMA-1_[3d7/FVO]_ and MSP-1_42[FVO]_. IgG fold changes were significantly higher after CHMI in the semi-immune participants compared with PfSPZ-CVac participants to all antigens (21/21; Figure 4), as well as to 6/21 antigens compared with the naïve participants (CSP, CelTOS, CyRPA-2, EBA-175, EXP-1, and MSP-1_42[3D7]_). At D28, IgG levels and fold-changes remained significantly higher in the semi-immune group versus the naïve group to 18/21 and 1/21 (RH1) antigens, respectively.

IgG subclasses were also significantly higher in the semi-immune group after CHMI compared with the naïve and PfSPZ-CVac volunteers for IgG1 (21/21), IgG2 (4/21; AMA-1_[3d7/FVO]_ and MSP-1_42[3d7/FVO]_), IgG3 (15/20; CSP, SSP-2/TRAP, AMA-1_[3d7/FVO]_, MSP-1_42[3d7/FVO],_ EBA-175, EXP-1, MSP-3, RH1, RH2, RH5, PTRAMP, CyRPA-1, and CyRPA-2), and IgG4 (8/18; CSP, AMA-1_[3d7/FVO]_, MSP-1_42[3d7/FVO]_, MSP-1_19,_ DBLα, and PTRAMP; Figure 3; Supplemental Table 2). At D28, the antibody levels of IgG subclasses remained similarly higher in the semi-immune individuals compared with the naïve individuals, in contrast to the IgM response at D28, which was higher in the semi-immune group compared with the naïve group only against RH5 (Supplemental Figure 3). The largest median differences (>4 log_10_ MFI) in antibody levels were observed in the semi-immune volunteers compared with the PfSPZ-CVac and naïve volunteers for IgG and IgG1 to AMA-1_[3d7/FVO]_ (Supplemental Table 2; Supplemental Figures 2 and 3).

#### Antibody response in the PfSPZ-CVac vaccine group by dose category.

**Figure 5.**
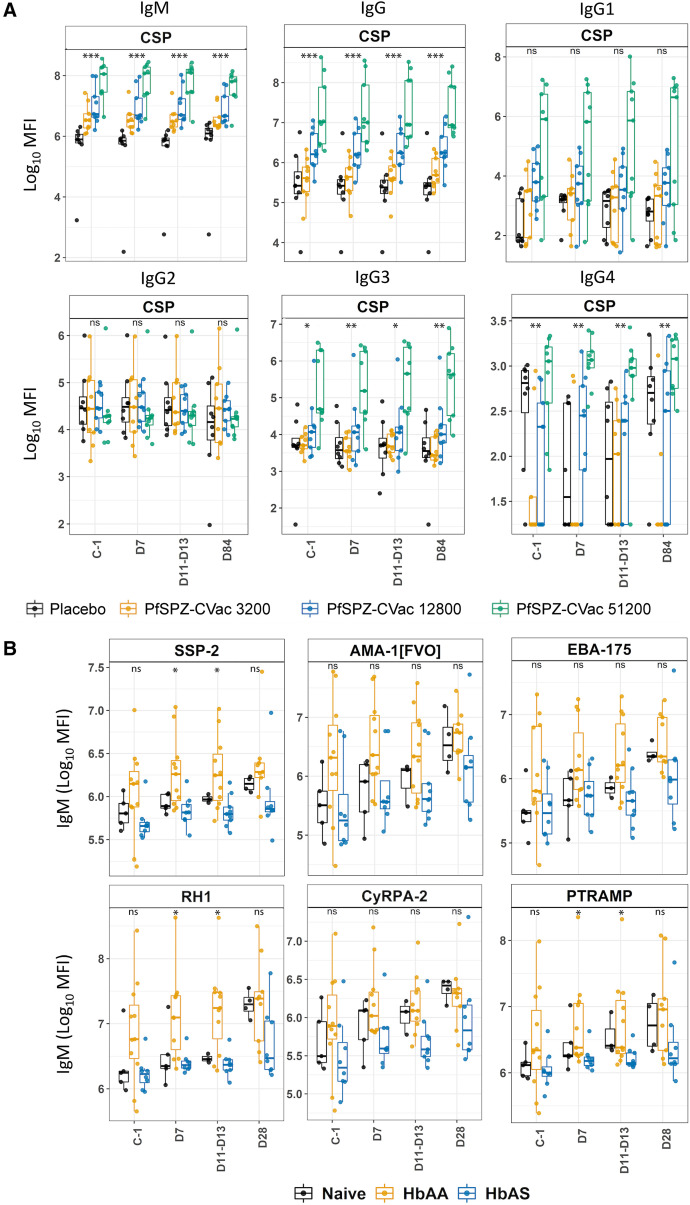
Antibody response in volunteers from the PfSPZ-CVac and semi-immune comparison groups. (**A**) IgM, IgG, and IgG isotype responses to CSP antigen in European malaria-naïve adult volunteers who received a placebo or three different doses of the PfSPZ-CVac. The *y*-axis in panel **A** reveals the log_10_ MFI of antibody levels to CSP antigen in the placebo, 3,200-dose, 12,800-dose, and 51,200-dose PfSPZ-CVac group categories. (**B**) IgM response to six *P. falciparum* antigens in semi-immune Gabonese adult volunteers with or without HbAS compared with European malaria-naïve volunteers (control), before and after PfSPZ challenge. The *y*-axis in panel **B** reveals the log_10_ MFI of IgM responses to six *P. falciparum* antigens in malaria-naïve (control) and semi-immune Gabonese volunteers with HbAA and HbAS. The *x*-axes in panels **A** and **B** reveal the time points studied: C-1; D7; D11–D13; D28. Boxplots: lower and upper lines represent Q1 and Q3 and reveal the IQRs (IQR = Q3–Q1); horizontal lines within boxes indicate medians (Q2); whisker lines correspond to the highest and lowest values no greater than 1.5 times the IQR; and dots represent individual data points. Data points beyond the whisker lines are outliers. *P*-values of the Kruskal–Wallis test used for comparisons among the PfSPZ-CVac vaccine group categories, as well as among the naïve, HbAA, and HbAS groups, are shown as follows: *P-value <0.05; **P-value <0.01; ***P-value <0.001. Results of post hoc Dunn’s multiple comparison tests are presented in Supplemental Tables 3 and 4. C-1 = 1–2 days before PfSPZ challenge; D7, D11–13, and D28 = 7, 11–13, and 28 days after PfSPZ challenge, respectively; HbAA = normal hemoglobin; HbAS = sickle cell-trait hemoglobin; IQR = interquartile range; log_10_ = logarithmic 10 scale; MFI = median fluorescence intensity; ns = no statistically significant difference; *P. falciparum* = *Plasmodium falciparum*; PfSPZ = *P. falciparum* sporozites; PfSPZ-CVac = PfSPZ chemoattenuated vaccine; Q = quartile.

Individuals vaccinated with PfSPZ-CVac had significantly higher IgM, IgG, IgG3, and IgG4 levels to CSP than placebo recipients (Figure 5A; Supplemental Table 4). Among vaccinees, CSP antibody levels at all timepoints were directly proportional to the PfSPZ-CVac dose received (i.e., 51,200 > 12,800 > 3,200), except for IgG2, the response to which appeared to have an inverse relationship with vaccine dose (Figure 5A). Between timepoints, the anti-CSP antibody levels exhibited a modest or no boost in all groups (Supplemental Table 4). Before challenge (C-1), compared with the placebo group, the 3,200- and 51,200-dose groups exhibited higher anti-SSP-2/TRAP IgM levels, and the 51,200-dose group exhibited higher anti-EXP-1 IgM and anti-AMA-1_[3d7]_ IgG levels but no significant differences in IgG1, IgG2, or IgG4 responses. After CHMI, significant differences between the vaccinated and placebo groups were maintained in addition to higher anti-CSP IgG4 levels in the 51,200-dose group compared with the placebo, 3,200-dose, and 12,800-dose groups (Figure 5A; Supplemental Table 4). No significant differences were found for IgG2 levels among these four groups at any time point; however, a trend of higher IgG2 levels to MSP-1_42[3d7]_ was observed in the 51,200-dose group compared with the 3,200- and 12,800-dose groups at D11–D13 (Supplemental Table 4). Among vaccinees, the 3,200-dose group had significantly higher levels of IgG4 to CyRPA-2 (C-1/D84) and IgG1 to RH5 (C-1/D7/D11–D13/D84) than the 51,200-dose group (Supplemental Table 4). The latter was the only significant difference found between these four groups for IgG1, in contrast to the significantly higher IgG1 levels observed in semi-immune compared with naïve and PfSPZ-CVac volunteers in the malaria pre-exposure group analysis.

#### Antibody response in the semi-immune group by hemoglobin status category.

Both the HbAA and HbAS semi-immune volunteers showed significantly higher antibody levels compared with the naïve (control) group, similar to what was observed in the semi-immune versus naïve groups in the malaria pre-exposure group analysis before and after CHMI. Among semi-immune volunteers, significant differences by hemoglobin status were observed for IgM and IgG4 responses post-infection. Compared with HbAA individuals, volunteers with HbAS had significantly lower levels of IgM to SSP-2/TRAP (D7/D11–D13/D19), LSA-1 (D7/D11–D13/D19), AMA-1_[FVO]_ (D19), RH1 (D7/D11–D13/D19), CyRPA-2 (D11–D13), PTRAMP (D7/D11–D13), and EBA-175 (D11–D13/D19); they also had significantly lower levels of IgG4 to EBA-175 (D11–D13) (Figure 5B; Supplemental Table 5). A trend of higher antibody levels was also observed in HbAS versus HbAA volunteers for IgG1 to AARP (D7) and PTRAMP (C-1), IgG3 to AARP (C-1/D7), and IgG4 to AMA-1_[3d7]_ (D7; Supplemental Table 5).

### Association of antibody levels with protection against malaria.

#### Malaria pre-exposure group (naïve, PfSPZ-CVac, and semi-immune).

In the univariable analyses, higher antibody levels to CSP at earlier timepoints were significantly associated with sterile protection against malaria diagnosed via TBS (microscopically patent parasitaemia; Supplemental Table 6) and qPCR (sub-patent parasitemia; Supplemental Table 7) for IgM, IgG, and IgG3. A protective trend against malaria diagnosed via TBS was observed for anti-DBLα IgG (C-1, D7, and D11–D13) and anti-RH5 IgG (C-1, D7, and D11–D13). A protective trend was also observed against malaria diagnosed via qPCR for anti-CSP IgG3 (C-1 and D11–D13), whereas anti-DBLα IgG4 (D11–D13) and anti-RH1 IgG3 (C-1, D7, D11–D13) were associated with risk of qPCR positivity. After adjusting for sex and malaria exposure (PfSPZ-CVac and semi-immune individuals compared with naïve individuals as a reference group), the associations were no longer statistically significant (not shown).

The geometric mean times to parasitemia (pre-patent periods) in volunteers who became malaria positive via TBS are shown in Table 2. Statistically significant results of univariable analysis on the association between antibody levels and pre-patent period and time to positive qPCR can be found in Supplemental Dataset 1. In the multivariable analysis at C-1, there were significant positive associations (β >0.0) with the pre-patent period for IgG to EXP-1, IgG1 to CSP and AMA-1_[3d7/FVO]_, IgG3 to CSP, AMA-1_[3d7/FVO]_, MSP-1_42[FVO]_, RH5, MSP-3, EXP-1, and DBLα, and IgG4 to AMA-1_[3d7]_ and EXP-1, but not for IgM or IgG2. There was also a significant positive association with the time to positive qPCR for IgG and IgG1 to EXP-1 and IgG3 to MSP-1_42[FVO]_, EXP-1, and DBLα (Supplemental Dataset 2). A significant negative association was found between IgG4 to DBLα at C-1 and time to positive qPCR. Most findings, such as the positive association of IgM levels with the prepatent period, were only significantly associated with semi-immune status (Supplemental Dataset 2), and fewer were associated with antibody levels.

**Table 2:** Proportion of thick blood smear-positive individuals and geometric mean of pre-patent periods within the malaria pre-exposure group (*n* = 61)

Parameters	Naïve (*n* = 14)	PfSPZ-CVac			Semi-Immune	
		3,200 PfSPZ-CVac (*n* = 9)	12,800 PfSZP-CVac (*n* = 9)	51,200 PfSPZ-CVac (*n* = 9)	HbAA (*n* = 11)	HbAS (*n* = 9)
TBS+, *n* (%)	14 (100)	6 (67)	3 (33)	0 (0)	7 (64)	5 (56)
GM PPP (days)	11.6	12.5	12.9	‒	16.9	19.1

GM = geometric mean; HbAA = normal hemoglobin genotype; HbAS = sickle cell trait hemoglobin genotype; PfSPZ = *Plasmodium falciparum* sporozoites; PfSPZ-CVac = immunization via direct venous inoculation of aseptic, purified, cryopreserved, non-irradiated PfSPZ to malaria-naïve, healthy adult volunteers taking chloroquine for antimalarial chemoprophylaxis; PPP = pre-patent period; TBS = thick blood smear.

**Figure 6.**
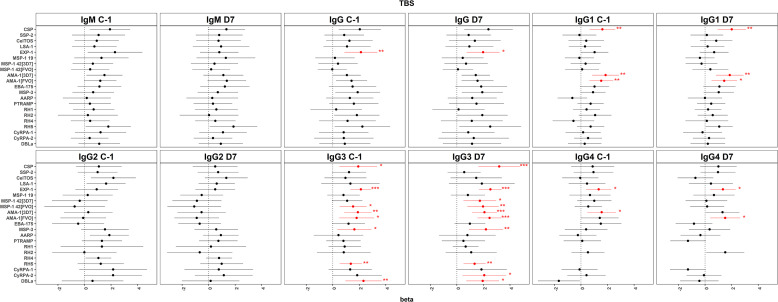
Forest plot of the multivariable regression models of antibody levels in relation to time to first positive malaria thick blood smear (pre-patent period). Horizontal lines represent the 95% CI of the beta coefficient in the regression for the following timepoints for IgM, IgG, and IgG subclasses: 1 to 2 days before *Plasmodium falciparum *sporozite (PfSPZ) challenge and 7 days after PfSPZ challenge. Red horizontal lines indicate significant results. The *y*-axis represents the antigens studied, and the *x*-axis represents the value of the beta coefficient. *P-value <0.05; **P-value <0.01; ***P-value <0.001.

After CHMI, in multivariable analysis on D7, significant positive associations with pre-patency remained for IgG to EXP-1, IgG1 to CSP and AMA-1_[3d7/FVO]_, IgG3 to CSP, AMA-1_[3d7/FVO]_, MSP-1_42[3d7/FVO]_, RH5, CyRPA-2, MSP-3, DBLα, and EXP-1, and IgG4 to AMA-1_[3d7/FVO]_ and EXP-1 (Figure 6; Supplemental Dataset 2), as well as for IgG3 to MSP-1_42[FVO]_ and IgG4 to RH2 on D11–D13 (Supplemental Dataset 2). On D7, there was a significant positive association between time to positive qPCR and antibody levels for IgG to EXP-1 and IgG3 to EXP-1, AMA-1_[FVO]_, and MSP-3, as well as for IgG3 to MSP-1_42[FVO]_ on D11–D13 (Supplemental Dataset 2). Additional analyses of antibody fold-changes (D7/C-1) in relation to patency are presented in Supplemental Table 8.

#### Lifelong malaria-exposed individuals (semi-immune with HbAA and semi-immune with HbAS).

Among lifelong malaria-exposed volunteers, 2/11 HbAA and 2/9 HbAS subjects remained TBS-negative and asymptomatic until D28 but were positive by qPCR. Among those who were qPCR-positive (16/20, 80%), 6/9 (67%) of HbAA and 1/7 (14%) of HbAS volunteers developed malaria symptoms. No significant associations were found between specific antibody responses and symptomatic malaria in these volunteers.

## DISCUSSION

The present study reveals significant differences in the type, magnitude, and dynamics of antibody responses among naïve, PfSPZ-CVac, and semi-immune volunteers before and after stringent standardized DVI inoculation of 3,200 PfSPZ (PfSPZ challenge) in three CHMI clinical trials performed in Europe and Africa. T cell-dependent antibody responses to a large number of antigen targets, particularly IgG1 and IgG3, were more efficient in controlling BS parasitaemia than TI (IgM and IgG2) or regulatory (IgG4) Ig, despite the prominent IgM secondary response observed in semi-immune volunteers. This finding is consistent with those of other CHMI studies on the importance for clinical immunity of the breadth of Fc-dependent “non-neutralizing” effector functions in cooperation with innate cells, which are largely mediated by cytophilic IgG subclasses.^38^ However, this finding is in contrast to other studies that revealed IgM responses associated with protection from infection and symptoms.^28^

Moreover, among the PfSPZ-CVac vaccine group, volunteers with lower protection (3,200 dose) after CHMI had a higher anti-CSP IgG2 response compared with those in the groups with higher protection. In addition, in the malaria pre-exposure group, semi-immune individuals (20% sterile protection) exhibited higher anti-CSP IgG4 levels at baseline than PfSPZ-CVac volunteers (67% sterile protection). Overall, the semi-immune group exhibited higher IgG2 (TI) and IgG4 (regulatory) responses to AMA-1 and MSP-1 than the other two groups, which might be linked to protection from symptomatic malaria.

Differences in antibody responses according to previous malaria exposure and future protection varied depending on the types and subclasses of Ig and the parasite stage of the target antigen. One of the most remarkable findings was the fast and significant increase in IgM levels against malaria BS antigens upon challenge in semi-immune individuals, mainly against merozoite antigens, revealing the hallmark of an IgM secondary memory recall response. This increase in Gabonese adults was stronger than the IgM secondary response in European vaccinees or the IgM primary response in naïve subjects during the first timepoints after CHMI. A different pattern was observed for IgM to sporozoite antigens, of which CSP is the prime vaccine target,^52^^–^^54^ whereby PfSPZ-CVac vaccinees exhibited significantly higher, dose-dependent, IgM levels against CSP and SSP-2/TRAP compared with semi-immune and naïve individuals before and after CHMI. This reflects 1) the much higher sporozoite and liver stage antigen load exposure in the PfSPZ-CVac volunteers (inoculated 8–10 weeks before CHMI)^13^ compared with continuous naturally occurring infections via female *Anopheles* mosquito injection in the skin (estimated 10–100 sporozoites)^55^^,^^56^ in semi-immune individuals, and 2) the waning of naturally acquired CSP IgM. The pattern of IgM responses observed in the present study does not follow established immunological canons, but the reasons and implications remain unclear because they have been much less studied than IgG responses.

One of the main producers of IgM in humans are the splenic marginal zone (MZ)-B cells (IgM^hi^IgD^low^CD1c^+^CD21^high^CD23^-^CD27^+^).^27^ Marginal zone B cells can be rapidly activated in a TI or TD manner by TI or TD antigens, toll-like receptor ligands, B cell activating factor, proliferation-inducing ligand, CD40 ligands, and IL-21, and can undergo class switch to IgG1, IgG2, and IgA2.^27^ Therefore, the intense inflammatory environment generated by malaria-infected red blood cells (RBCs) streaming into the spleen could explain the activation of MZ-B cells during malaria infection.^32^^,^^33^^,^^57^ Likely, *P. falciparum*-specific IgM^+^ MBCs remain residents of the spleen for life and engage at each malaria episode, generating a new batch of short-lived IgM^+^ plasma cells, and new waves of *P. falciparum*-specific IgM, as observed in semi-immune participants studied herein. In *P. falciparum*-exposed individuals from Papua New Guinea, it was found that circulating MZ-like B cells^58^ were decreased compared with nonexposed individuals^59^^,^^60^ and that a proportion of them were also IgG^+^.^59^ This finding could reflect migration to the spleen or apoptosis, as well as the class-switch recombination capacity of MZ-B cells.

Although it is possible that not all B cells follow the canonical switching order (IgG3, IgG1, IgG2, and IgG4),^24^ the study data reflect this well because the predominant subclass in the PfSPZ-CVac participants (recently exposed to *P. falciparum* parasites) was IgG3, whereas IgG1, IgG2, and IgG4 exhibited few significant differences compared with naïve and semi-immune volunteers. However, in the semi-immune group (lifelong exposure), IgG1 was the most prominent subclass before and after CHMI, followed by IgG3, IgG4, and IgG2. The importance of cytophilic antibodies for anti-parasite immunity was confirmed in the current study by their significant association with longer pre-patent periods for IgG1 and IgG3 to several antigens, which is in agreement with previous studies.^61^ In addition, the tolerogenic response in the semi-immune group was made evident by the significantly higher levels of IgG2 and IgG4 to several BS antigens and CSP compared with naïve and PfSPZ-CVac volunteers. In the PfSPZ-CVac group, volunteers who received the highest and 100% protective dose (51,200) exhibited significantly higher IgG4 levels to CSP than the placebo, 3,200-dose, and 12,800-dose groups after CHMI. IgG4 is produced in a TD manner after exposure for an extended period of time to a protein or glycoprotein antigen (frequently in the absence of an ongoing infection)^62^ and class switching seems to be driven by CD4^+^ T helper cells expressing IL-10.^62^ Hence, the higher levels of IgG4 to CSP in the semi-immune group compared with PfSPZ-CVac participants could hamper an effective pre-erythrocytic response after CHMI with PfSPZ and partially explain the lower rates of sterile protective immunity in Gabonese volunteers compared with PfSPZ-CVac, as well as the lower vaccine efficacy of whole PfSPZ vaccines or CSP subunit vaccines in malaria-exposed individuals compared with naïve populations.^14^^,^^16^^,^^63^ Nevertheless, IgG4 is the least abundant in serum and did not seem to impede sterile protection by the 51,200-dose PfSPZ-CVac. Likely, the orchestrated humoral and cellular responses, differences in antibody magnitudes between IgG subclasses, and competition of cytophilic and non-cytophilic IgG for binding to epitopes, finally determine resistance or susceptibility to malaria infection.^39^

On the other hand, IgG2 antibodies are generated in a TI manner, like IgM, and are associated with chronic inflammation in diseases such as sarcoidosis and Crohn’s disease, or in the case of falciparum malaria, in response to prolonged pathogenic stimulation.^24^ Consequently, levels of IgG2 to CSP exhibited a direct relationship with malaria infection in the PfSPZ-CVac vaccine group.

Examining the patterns of antibody response in the semi-immune HbAS subjects (who are naturally partially protected against *P. falciparum* infection and symptoms) could shed some light on the determinants and role of these antibodies in malaria. Infected HbAS volunteers (with less prominent IgM responses [TI]) exhibited 1) lower rates of symptomatic malaria (14% versus 67%), 2) longer pre-patent periods than infected HbAA semi-immune volunteers (Table 2), although the differences in geometric means were not statistically significant,^43^ and 3) a trend toward higher IgG1, IgG3, and IgG4 levels (TD) for some antigens. It appeared that when only total IgG was measured, the IgG subclass differences between HbAS carriers and HbAA semi-immune individuals could not be captured because fewer differences were found in total IgG.

Several mechanisms have been proposed to explain HbAS malaria protection, mainly attributed to enhanced removal of parasitized RBCs and less cytoadherence.^64^ Phagocytosis by human macrophages of ring-parasitized but not trophozoite-parasitized RBCs is selectively enhanced in mutant (HbAS) versus normal RBCs.^64^ Phagocytosis of ring-infected RBCs can be repeated without loss of function, whereas the digestion of mature malaria parasite stages containing hemozoin affects important functions of monocytes.^64^ Therefore, it is possible that HbAS carriers control BS parasites by abrogating (at least partially) the development of rings into schizonts, reducing parasite growth and density. Early control of BS infections could lead to lower erythrocytic parasite exposure and less inflammation. The latter would hypothetically decrease the activation of MZ-B cells in the spleen of HbAS individuals, possibly explaining the discrete lower IgM levels compared with the HbAA semi-immune volunteers in the present study. Interestingly, in a recent CHMI in semi-immune volunteers, IgM binding to *P. falciparum* merozoites exhibited the weakest association with a longer time to treatment, whereas IgG1 binding exhibited the strongest association.^38^

Regarding translational implications, lifelong malaria-exposed individuals were found to have a regulatory antibody response (IgG4) to CSP, the target of current malaria vaccines. Additionally, the antibody profile of semi-immune individuals with normal HbAA compared with HbAS individuals (naturally protected) revealed a modest but significant bias toward a higher TI response (IgM) and a trend toward lower TD and cytophilic antibodies against some *P. falciparum* antigens, possibly due to their higher exposure to BS, which diminishes the control of infection, reflected in shorter pre-patent periods and more cases of symptomatic malaria. In agreement with this finding, anti-malarial prophylaxis improves CD4^+^ T cell function and limits IL-10 production.^65^

The main limitations of the present study are the small sample sizes of the groups compared and the absence of Fc-dependent functional assays to test some of the hypotheses. Nevertheless, the results are aligned with those of other studies regarding the correlation of Fc-mediated function with cytophilic antibodies in clinical immunity, as well as the lower association of IgM levels with time to treatment compared with other immunoglobulins.^38^ The strengths of the current study lie in the highly controlled conditions of CHMI that facilitate the identification of the exact parasite inoculum and moment of infection, better comparisons between individuals, and the wide array of antigens tested, as well as the study of six different Ig, allowing for the characterization of TD and TI responses to CHMI with PfSPZ Challenge.

## CONCLUSION

The PfSPZ-CVac induces strong pre-erythrocytic antibody immunity, whereas lifelong natural malaria exposure elicits intense and broad responses to BS antigens, with prolonged pre-patent periods and fewer malaria symptoms after CHMI than experimental immunization. Although the protective role of TD cytophilic IgG subclasses in malaria immunity has been confirmed, it remains to be elucidated whether the IgM response in lifelong exposed individuals is the desired humoral response because this is most likely TI. It is nevertheless important and perhaps inevitable in natural immunity, conferring protection against infection and malaria symptoms.^28^^,^^66^^,^^67^ It is possible that IgM TI responses are compensating for a defective TD response caused by repeated exposure to malaria parasites because it is less prominent in HbAS volunteers naturally protected from malaria. To better understand *P. falciparum*-specific TI responses, the authors of future malaria NAI and vaccine studies should always measure IgM and IgG subclasses in individuals of diverse ages, exposures, timings of malaria infection, and endemic settings. Next-generation vaccines should be designed to trigger strong specific cytophilic antibody responses while accounting for less effective TI responses linked to malaria parasitemia and their potential impact on vaccine immunogenicity.

## Supplemental Materials

10.4269/ajtmh.25-0384 Supplemental Materials
