# Supplementary material for: IgM, IgG, and IgG Subclass Antibody Responses to Plasmodium falciparum Proteins in Naïve, Malaria-Vaccinated and Semi-Immune Volunteers after Controlled Human Malaria Infection
Source: Am J Trop Med Hyg. 2025 Sep 30;113(6):1235–48. doi: 10.4269/ajtmh.25-0384 (PMC12676597; doi:10.4269/ajtmh.25-0384)
Supplement: Supplemental Materials [file tpmd250384.SD1.pdf]

SUPPLEMENTARY FIGURES

Manuscript title: "*IgM, IgG and IgG subclass antibody responses to Plasmodium falciparum proteins in naïve, malaria-vaccinated and semi-immune volunteers after controlled human malaria infection*." (Gómez-Pérez et al).

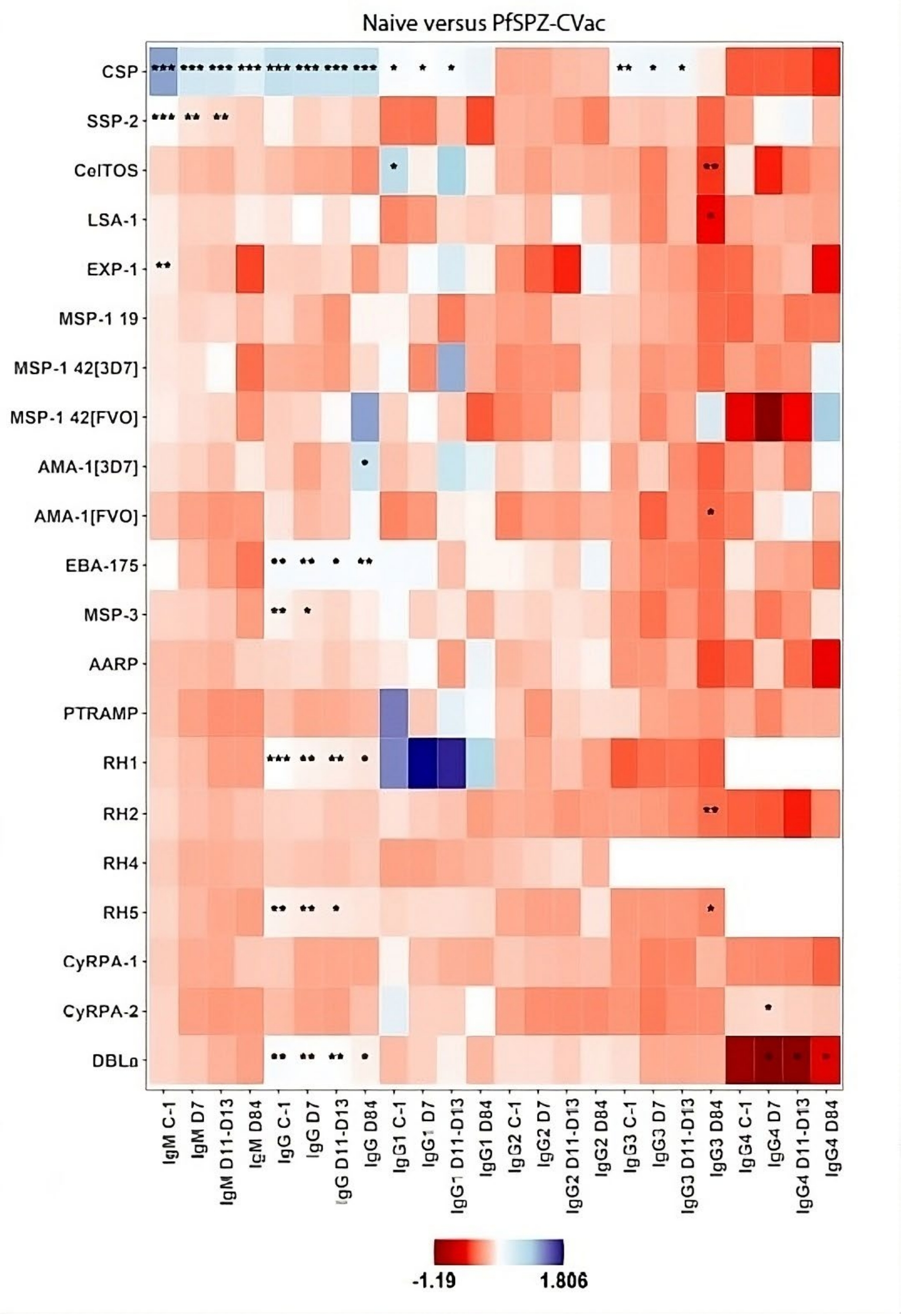

Figure S1. Heatmap analysis of the difference in antibody levels between PfSPZ-CVac and Naïve volunteers measured at baseline (C-1, before PfSPZ Challenge), and seven days (D7), 11-13 days (D11-13), and 84 days (D28) after PfSPZ Challenge. Columns represent the difference in antibody levels in $\log_{10}$ MFI between the two groups (PfSPZ-CVac - Naïve) per isotype and timepoint (C-1, D7, D11-13 or D84), and the rows represent the *P. falciparum* antigens studied. The differences in antibody levels are color coded from red (towards negative difference: PfSPZ Vaccine < Naïve) to blue (towards positive difference: PfSPZ-CVac > Naïve); the white color (cut off for color coding) represents a difference in antibody level = 0.308. The *P*-values of the Dunn test paired comparisons are shown as follow: *, *P*-value ≥ 0.05 and < 0.10; **, *P*-value < 0.05; ***, *P*-value < 0.01. Results of post hoc Dunn's test are also presented in Table S2.

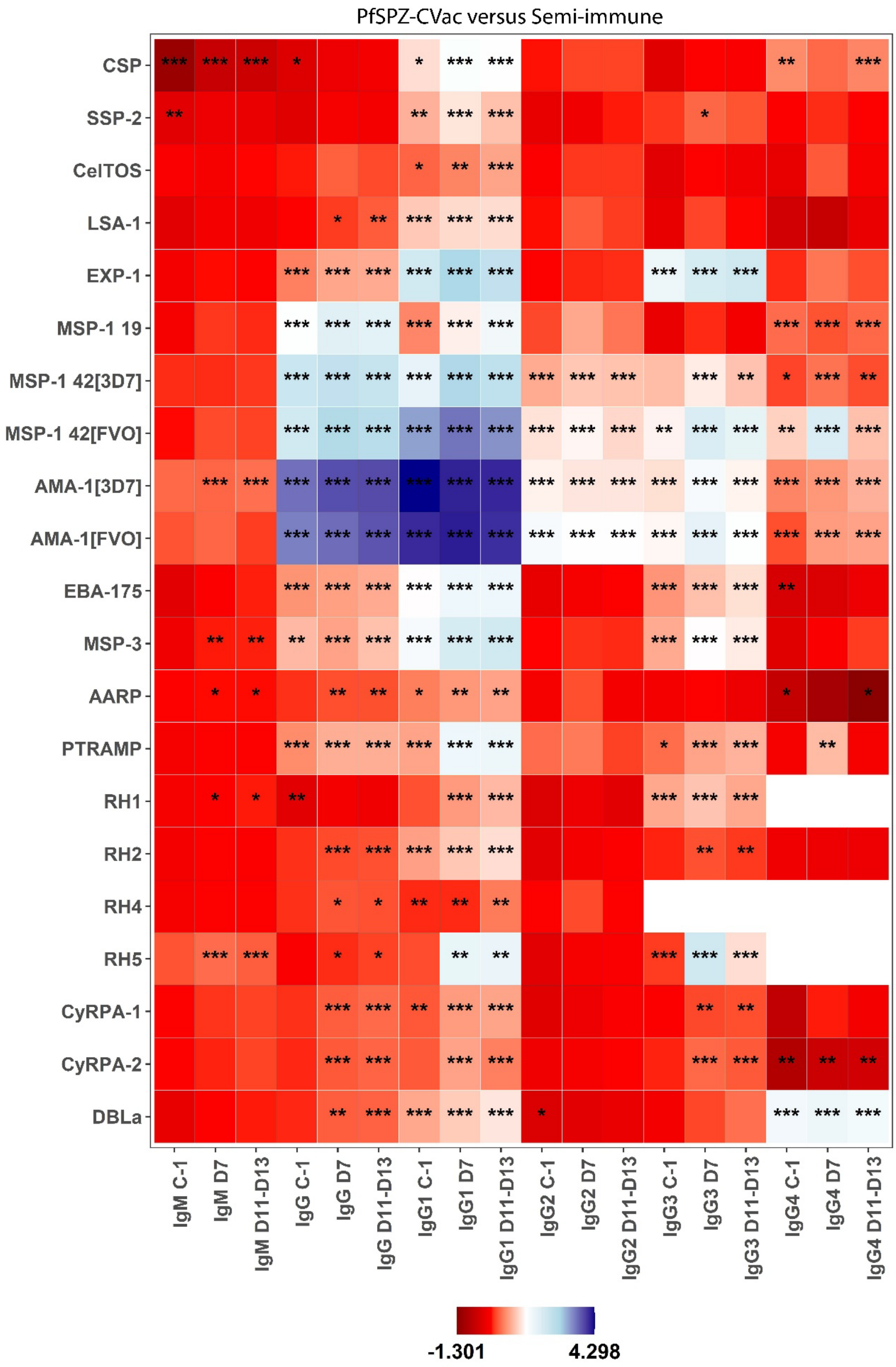

PfSPZ-CVac versus Semi-immune
CSP
SSP-2
CelTOS
LSA-1
EXP-1
MSP-1 19
MSP-1 42[3D7]
MSP-1 42[FVO]
AMA-1[3D7]
AMA-1[FVO]
EBA-175
MSP-3
AARP
PTRAMP
RH1
RH2
RH4
RH5
CyRPA-1
CyRPA-2
DBLa
IgM C-1
IgM D7
IgM D11-D13
IgG C-1
IgG D7
IgG D11-D13
IgG1 C-1
IgG1 D7
IgG1 D11-D13
IgG2 C-1
IgG2 D7
IgG2 D11-D13
IgG3 C-1
IgG3 D7
IgG3 D11-D13
IgG4 C-1
IgG4 D7
IgG4 D11-D13
-1.301
4.298

Figure S2. Heatmap analysis of the difference in antibody levels between Semi-immune and PfSPZ-CVac volunteers measured at baseline (C-1, before PfSPZ Challenge), and seven days (D7), 11-13 days (D11-13), and 28 days (D28) after PfSPZ Challenge. Columns represent the difference in antibody levels in $\log_{10}$ MFI between the two groups (Semi-immune - PfSPZ-CVac) per isotype and timepoint (C-1, D7, D11-13 or D28), and the rows represent the *P. falciparum* antigens studied. The differences in antibody levels are color coded from red (towards negative difference: Semi-immune < PfSPZ-CVac) to blue (towards positive difference: Semi-immune > PfSPZ-CVac); the white color (cut off for color coding) represents a difference in antibody level = 1.499. The *P*-values of the Dunn test paired comparisons are shown as follow: *, *P*-value ≥ 0.05 and < 0.10; **, *P*-value < 0.05; ***, *P*-value < 0.01. Results of post hoc Dunn's test are also presented in Table S2.

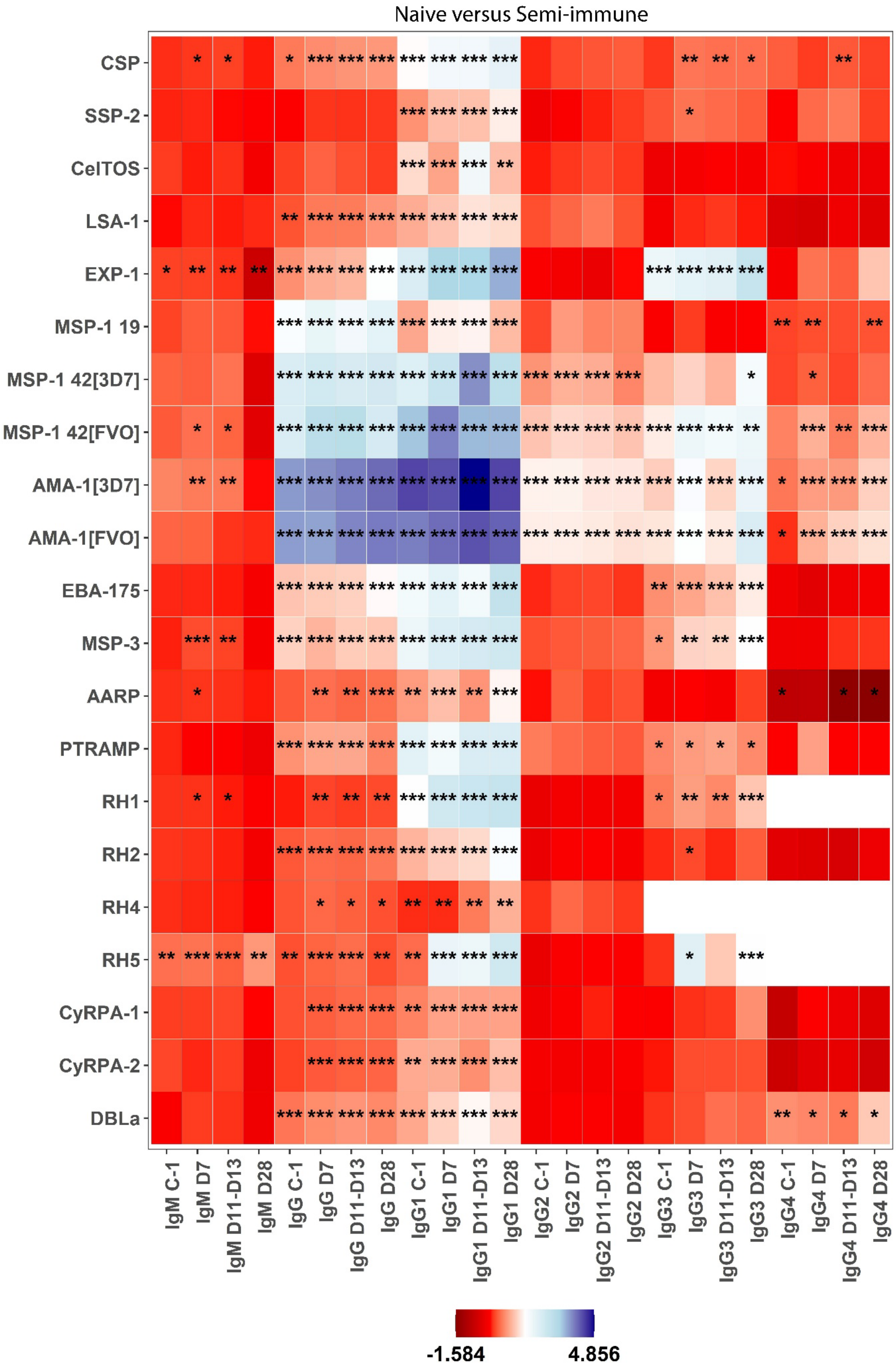
Naive versus Semi-immune
CSP
SSP-2
CelTOS
LSA-1
EXP-1
MSP-1 19
MSP-1 42[3D7]
MSP-1 42[FVO]
AMA-1[3D7]
AMA-1[FVO]
EBA-175
MSP-3
AARP
PTRAMP
RH1
RH2
RH4
RH5
CyRPA-1
CyRPA-2
DBLa
IgM C-1
IgM D7
IgM D11-D13
IgM D28
IgG C-1
IgG D7
IgG D11-D13
IgG D28
IgG1 C-1
IgG1 D7
IgG1 D11-D13
IgG1 D28
IgG2 C-1
IgG2 D7
IgG2 D11-D13
IgG2 D28
IgG3 C-1
IgG3 D7
IgG3 D11-D13
IgG3 D28
IgG4 C-1
IgG4 D7
IgG4 D11-D13
IgG4 D28
-1.584
4.856

Figure S3. Heatmap analysis of the difference in antibody levels between Semi-immune and Naïve groups measured at baseline (C-1, before PfSPZ Challenge), and seven days (D7), 11-13 days (D11-13), and 28 days (D28) after PfSPZ Challenge. Columns represent the difference in antibody levels in $\log_{10}$ MFI between the two groups (Semi-immune - Naïve) per isotype and timepoint (C-1, D7, D11-13 or D28), and the rows represent the *P. falciparum* antigens studied. The differences in antibody levels are color coded from red (towards negative difference: Semi-immune < Naïve) to blue (towards positive difference: Semi-immune > Naïve); the white color (cut off for color coding) represents a difference in antibody level = 1.636. The *P*-values of the Dunn test paired comparisons are shown as follow: *, *P*-value ≥ 0.05 and < 0.10; **, *P*-value < 0.05; ***, *P*-value < 0.01. Results of post hoc Dunn's test are also presented in Table S2.
