# Supplementary material for: IgM, IgG, and IgG Subclass Antibody Responses to Plasmodium falciparum Proteins in Naïve, Malaria-Vaccinated and Semi-Immune Volunteers after Controlled Human Malaria Infection
Source: Am J Trop Med Hyg. 2025 Sep 30;113(6):1235–48. doi: 10.4269/ajtmh.25-0384 (PMC12676597; doi:10.4269/ajtmh.25-0384)
Supplement: Supplemental Materials [file tpmd250384.SD2.pdf]

Supplementary Table S1: Glossary of antigens for Luminex assays

Manuscript title: "*IgM, IgG and IgG subclass antibody responses to Plasmodium falciparum proteins in naïve, malaria-vaccinated and semi-immune volunteers after controlled human malaria infection*." **(**Gómez-Pérez et al).

***Plasmodium falciparum* pre-erythrocytic stage antigens**	
**CSP**	Circumsporozoite protein is a major surface protein that forms a dense coat on the sporozoite's surface and is critical for sporozoite function and invasion of hepatocytes. Structurally, CSP is divided into three regions: (1) the NH_2-terminal region, (2) a centrally-located repeat region (immunodominant), and (3) the COOH-terminus which contains a type I thrombospondin repeat region (Noe AR *et al*. Plos One 2014). During the liver stage, the parasite introduces CSP into the hepatocyte cytoplasm and a nuclear localization signal to enter its nucleus. Inside the hepatocyte nucleus, CSP down regulates several host genes including those involved in inflammation and metabolic processes to favor parasite growth. The presence of CSP in the hepatocyte has shown to enhance parasite growth of the liver stages in vitro and in vivo (Singh AP *et al.* Cell 2007).
**SSP-2 (TRAP)**	Sporozoite surface protein 2 was first described in *P. yoelii* sporozoites and later on identified as *P. falciparum* TRAP (thrombospondin-related adhesive protein). SSP2/TRAP transcripts have been found in both sporozoites and infected hepatocytes (Bodescot M *et al*. Parasitol Res 2004). Human volunteers immunized with irradiated sporozoites and

protected against malaria develop antibody and proliferative T-cell responses to PfSSP2 (Rogers WO *et al*. Proc Natl Acad Sci U S A 1992)

**CelTOS**	Cell-traversal protein for ookinetes and sporozoites. This protein plays an important role in cell transversal of host cells in both mosquito and vertebrates and is required for successful malaria infections. CelTOS is highly conserved among the *Plasmodium* species, and therefore can be cross-protective between species. The biological activity of CelTOS-specific antibodies against the malaria parasite is likely linked to the impairment of sporozoite motility and hepatocyte infectivity (Bergmann-Leitner ES *et al.* Plos One 2010).
**LSA-1**	Liver Stage Antigen-1. Shortly after hepatocyte invasion, the parasite starts to produce LSA-1, which accumulates within the parasitophorous vacuole surrounding the mass of developing merozoites. LSA-1 is one of the few antigens known to be specifically expressed during the liver stage of *P. falciparum*. Studies of human immunity following exposure to radiation-attenuated sporozoites, as well as exposure to naturally transmitted parasites, have associated protection with a specific LSA-1 immune response (Nicoll WS *et al*. Malar J. 2011).

***Plasmodium falciparum* liver and blood-stage antigens**

**AMA-1**	Apical membrane antigen-1 is a type I integral membrane protein, the extracellular region of which consists of three domains based on the connectivities of its eight intramolecular disulfide bonds: an N-terminal domain I, a central domain II, and a C-terminal domain III. AMA-1 interacts with RON2 (a parasite protein) to form a protein complex that plays a key role in the invasion of host cells by malaria parasites (Lim SS *et al.* Biochemistry 2014). Inhibition of AMA1-RON2

interaction has shown to block merozoite invasion (Srinivasan P *et al*. Nat Commun 2013). There are amino acid polymorphisms in its primary sequence, generating various genotypes (3D7 and FVO, among others, which have 24 amino acid differences). Antisera to 3D7 were more strain-specific than antisera to FVO (Lim SS *et al.* Biochemistry 2014) in the preclinical studies. A vaccine based on AMA1 3D7 appeared to induce strain-specific immunity (Mahamadou 2011 NEJM).

EXP-1 Exported protein 1. Also called circumsporozoite-related antigen, QF116 antigen, or antigen 5.1, is a 23-KDa protein expressed in liver stage parasites and asexual blood stages and secreted by the parasite into de host cell (Sanchez GI *et al.* Exp Parasitol 1994). It is an integral membrane protein found in the membrane of the parasitophorous vacuole and within vesicles in the infected red blood cell cytoplasm. It is believed to play a role in the trafficking of parasite proteins. EXP-1 leaves the parasite itself trespassing the membrane of the parasitophorous vacuole inside a vesicle, traversing the host cytoplasm to finally be inserted in the red blood cell plasmalema (Simmons D *et al.* EMBO J 1987). EXP-1 is highly conserved, suggesting that it either has an important function in the parasite or that it is not under immune pressure (Meraldi V *et al.* Parasite Immunol 2004). Immune response against the malaria EXP-1 protein may result in HTLV-I-cross-reacting antibodies that can lead to false-positive immune assays (Porter KR *et al.* Clin Diagn Lab Immunol 1998).

MSP-1 Merozoite surface protein-1 is a major surface protein and the most abundant merozoite surface component. It is GPI-anchored and synthesized as an ∼200 kDa protein that in *P. falciparum* associates with at least two other peripheral proteins belonging to the MSP-3 and MSP-7 families (Das S *et al*. Cell Host Microbe 2015). At the time of merozoite invasion, the 42 kDa of merozoite surface protein-1 (**$MSP\text{-}1_{42}$**) is proteolytically cleaved into a soluble fragment of 33 kDa ($MSP\text{-}1_{33}$) and a 19 kDa fragment (**$MSP\text{-}1_{19}$**) that remains on the merozoite surface and is carried into newly invaded erythrocyte. **$MSP\text{-}1_{42}$** has amino acid polymorphisms generating different genotypes like **3D7**-type and the **FVO**-type**.** Antibodies to $MSP\text{-}1_{19}$ are important in preventing erythrocyte invasion (Nurul AA *et al*. Trop Biomed 2010). $MSP\text{-}1_{42}$ vaccines however were not sufficiently immunogenic to confer protection against clinical malaria in 12–47 month-old children in Kenya (Ogutu BR *et al.* Plos One 2009). MSP-1 has high affinity for red blood cell membranes, and has been found to bind to erythrocyte glycophorin A (GYPA), Band 3, heparin-like molecules, and into the inner red blood cell membrane skeleton protein spectrin (Herrera S *et al*. EMBO J. 1993; Das S *et al*. Cell Host Microbe 2015).

***Plasmodium falciparum* blood-stage antigens**

**MSP-3**	Merozoite surface protein-3. It is a non GPI-anchored surface protein that is soluble and present on the merozoite surface as a protein complex, possible through protein-protein interactions. Anti-MSP-3 antibodies were found to mediate antibody-dependent cellular inhibition of the parasite (Druilhe P *et al.* PLoS Med 2005) This protein forms oligomers, although it is a soluble protein, and it can bind to heme. However, the significance of these characteristics is not well understood (Imam M *et al*. J Biol Chem 2014).
**EBA-175**	Erythrocyte Binding Antigen-175 has an important role in the invasion of human erythrocytes. It mediates adhesion to erythrocytes through binding of the Duffy-binding-like (DBL) domains in its extracellular domain to Neu5Acα2-3Galactose displayed on the O-linked glycans of Glycophorin-A (GYPA, the major glycoprotein found on human erythrocytes). However, some studies show that regions outside of the DBL domains are also important for interactions with GYPA (Wanaguru MK *et al.* J Biol Chem 2013)
**AARP**	Asparagine rich parasite protein. This protein harbors a predicted signal sequence, a C-terminal transmembrane region and whose transcription and translation patterns are similar to some well characterized merozoite surface/apical proteins. It is localized in the apical ends of the merozoite rhoptries. It binds to erythrocytes through its N-terminal region with a receptor on the erythrocytes surface that is sensitive to trypsin and neuraminidase treatments suggesting a role of AARP in erythrocyte binding and invasion by the merozoite. The N-terminal region is highly conserved (Wickramarachchi T et al., Plos One 2008)

PTRAMP *Plasmodium* thrombospondin-related apical merozoite protein. This protein localises in the apical organelles of merozoites within intraerythrocytic schizonts. PTRAMP co-localises with AMA-1 in developing micronemes and subsequently relocates onto the merozoite surface. Although the gene appears to be specific to the *Plasmodium* genus, orthologues are present in the genomes of all malaria parasite species examined suggesting a conserved function in host-cell invasion (Thompson J et al., Mol Biochem Parasitol 2004).

RH1 Reticulocyte Binding Protein Homologue 1, is a sequence polymorphism of the RH protein family involved in erythrocyte invasion (Taylor HM et al., Infect. Immun. 2002).

RH2 Reticulocyte Binding Protein Homologue 2.

RH4 Reticulocyte Binding Protein Homologue 4.

RH5 Reticulocyte Binding Protein Homologue 5 is a member of the super family of erythrocyte ligands referred to as the Reticulocyte Binding Like proteins (RBLs). RH5 differs from the other members of the RH family in that it is much smaller and the gene encoding RH5 is refractory to genetic deletion in all tested strains, implying that it is essential for blood stage growth. RH5 binds erythrocytes and is implicated in the species tropism of erythrocyte invasion. Its receptor is basigin/CD147, and importantly, unlike all other known erythrocyte–merozoite receptor–ligand interactions, the basigin-RH5 interaction appears to be essential for erythrocyte invasion in every strain tested (Bustamante LY *et al*. Vaccine 2013). It interacts with Pf proteins Ripr (PfRH5-interacting protein) and CyRPA (see below) forming a multiprotein complex on the merozoite surface (Reddy KS *et al.* Proc Natl Acad Sci U S A. 2015).

**CyRPA**	Cysteine-rich protective antigen is an interacting partner of RH5-Ripr that fastens the RH5/PfRipr/CyRPA multiprotein complex on the merozoite surface. CyRPA is GPI- anchored, localized in the micronemes, and essential for erythrocyte invasion. (Reddy KS *et al.* Proc Natl Acad Sci USA. 2015). The stage-specific expression of CyRPA in late schizonts resembles that of proteins known to be involved in merozoite invasion (Dreyer AM *et al.* J. Immunol 2012).
**DBLα**	Duffy Binding-like domain sequence class alpha. The DBL domains are common adhesion molecules present in *P. falciparum* erythrocyte membrane protein 1 (PfEMP1) variants, which are responsible for immune evasion and cytoadherence (Mayor A *et al.* Infect Immun 2009). PfEMP1-DBLα is a major parasite ligand and has been shown to contribute to rosette formation (Kalambaheti HN *et al.* Malar J 2009).
**Negative control antigen**	
**BSA**	Bovine serum albumin
